# Impact of both early-age acclimation and linseed dietary inclusion on fat deposition and fatty acids’ meat traits in heat-stressed broiler chickens

**DOI:** 10.5455/javar.2021.h508

**Published:** 2021-06-19

**Authors:** Bengharbi Zineb, Dahmouni Said, Benabdelmoumene Djilali

**Affiliations:** Laboratoire de physiologie animale appliquée, FSNV-UMAB, Mostaganem, Algeria

**Keywords:** Broilers, heat stress, meat quality, linseed, fatty acids

## Abstract

**Objective:**

The purpose of this work was to investigate the combination of early-age acclimation and linseed dietary inclusion in enriching polyunsaturated fatty acids (PUFAs) in broilers’ meat as a strategy to mitigate heat stress.

**Materials and Methods:**

A total of 400 broiler chicks were assigned to four experimental groups with four duplicates (25 animals each): C: control (basal diet), AC: early-age acclimated (basal diet), Cl: fed 5% ground linseed, and Acl: early-age acclimated and fed 5% ground linseed. The lipid and fatty acid contents of different parts (breast, thigh, liver, subcutaneous, and abdominal fat) of broilers were determined.

**Results:**

Low levels of lipids and unsaturated fatty acids have been found in the meat of acclimated broilers. Higher levels of linolenic acids were noted in Cl thigh meat compared to C (6% *vs.* 2.68%, respectively). The results showed that oleic and linoleic acids constitute a large part of the PUFAs of different meats. The most elevated levels of monounsaturated fatty acids were recorded in the breast meat of AcL animals. The highest content of omega-3 was recorded in the liver of AcL animals compared to that of C (14.98% *vs.* 7.8%, respectively).

**Conclusion:**

We suggest that the combination of treatments during hot conditions has led to the reversion of the environment-affected variables to accepted values, and yields better thermoresistance, PUFA-enriched meat, and safeguard animal health which conferred to birds’ better solutions to reduce fatigue and hypoxic activities, which induces a considerable consumption of oxygen.

## Introduction

Poultry products have become, for low-income populations, the most affordable source of protein and polyunsaturated fatty acids (PUFAs). The accretions in broilers’ meat is related to the alteration of feeding diets. The chicken industry is in continuous development to meet the increasing demands of consumers [1-3], where, until 2025, the yearly growth rate of poultry protein is expected to reach 2.4% [4].

To consider the changes, enhancements have been concentrated principally on growth rate selection, feeding conversion efficacy, and the level of muscularity, leading to substantial changes in broilers [4,5]. Over the past 50 years, optimizing feed producing a pound of meat has reduced the required time to raise animals by 50%. Despite the rising demand for poultry products worldwide, consumption levels remain relatively low in hot climate regions [6]. The extension of poultry farming in the hot areas is subjected to several restrictions; the most evident of which is the hot climate [7]. Commercial poultry is well known to suffer from temperature elevation [8,9].

Thermal stresses can occur as acute and chronic according to the duration and the degree of severity [10-12]. Lu et al. [10] have reported that prolonged heat-related stress can impair meat quality by influencing aerobic metabolism, glycolysis, and triglycerides deposition in the liver [13], leading to less consumer suitability [14] due to pale meat color [12] with decreased water-holding capacity and increased cook and drip losses [12,15].

Fatty acids and cholesterol levels in meat are of great importance due to their implications in human health [16,17]. Ratios of PUFA/ saturated fatty acid (SFA) and n-−6/n-−3 ratios reveal the nutritional value of fats. During the last decades, research objectives were oriented toward lipid metabolism, particularly the influence of single fatty acids on preventing coronary heart diseases. In addition, conjugated linoleic acid (CLA) is considered very important due to its anticarcinogenic activity and the CLA/CLA + cholesterol ratio [18].

Rearing in high ambient temperatures alters the metabolism of broilers, leading to increased lipid depositions and a higher proportion of adipose tissues [19]. This is also associated with the elevation of SFA levels. A high proportion of saturated fats in the diet has been of great concern since their over-consumption increases the risk of heart diseases. Furthermore, monounsaturated fatty acids (MUFAs) and PUFAs, particularly *n*−3 PUFA, have a beneficial role in reducing human blood cholesterol levels. However, due to its beneficial impact on human health in enhancing broiler meat with *n*−3 fatty acids, flaxseeds have been widely exploited as a vital source of *α*-linolenic acid [20,21].

Over the last six decades, many potential methods to alleviate heat stress in birds were studied and encouraging results were found. Some studies targeted feeding strategies (feed restriction, double of feeding regime, or wet feeding). Other works tried dietary fat inclusion or supplementation of vitamins (A, C, and E), minerals, and electrolytes. However, many authors investigated the dietary supplementation of phytochemicals (lycopene, resveratrol, epigallocatechin gallate, or curcumin) to counter the detrimental effects of heat-related stress on poultry health and hot performance environment [3].

As it contains 35%–45% oil, which is more than 70% *α*-linolenic acid, linseed is considered an essential source of fat in animal feeds, especially in *n*−3 fatty acid meat enrichment. This plant also contains many nutritional properties (metabolizable energy, lignans, protein content, and dietary fibers) [22]. Dietary linolenic acid can be physiologically synthesized as a precursor into other beneficial *n*−3 PUFAs; therefore, reducing carcass body fat composition can be influenced by dietary PUFAs in poultry [23,24].

In previous work, we studied the synergistic influence of early-age thermal conditioning (TC) of chicken broilers, and the supplementation of 5% flaxseed (*Linum usitatissimum *L.) in experimental animals feed on carcass quality yield and weight development of different organs at different ages [7,9].

Therefore, the objective of the present research study was to highlight the thermal acclimation of broiler chicks and linseed supplemented diet effects to counter the adverse impacts of heat stress and improve meat traits by MUFA incorporation. Thus, the lipid deposition and fatty acid profiles of five parts of the carcass in broilers (abdominal fat, breast, liver, subcutaneous, and thigh) were investigated.

## Materials and Methods

### Ethical approval

All experiments were carried out in compliance with the guidelines set by the Algerian Association of Experimental Animal Sciences (No. 45/DGLPAG/DVA.SDA.14).

### Study protocol

A total of 400 mixed-sex chicks (ISA Hubbard) aged 1 -day-old were purchased from a local commercial hatchery. Depending on TC and linseed-supplemented [9] feeding diet, the chicks were randomly divided into four groups [6,7]: non-treated control (C), acclimated (Ac), CL, and AcL. Standard and supplemented diets were isocaloric [metabolizable energy (ME): 2887.06 kcal/kg]. Feedstuff and levels of ME of different age diets (starter, grower, and linseed) were analyzed and calculated [25]. Experimental birds were bred (according to European legislation for the protection of animals used for scientific purposes) at the Mostaganem University poultry station.

### Animals’ sacrificing and sampling

Twenty birds of each group were slaughtered (in conformance with the approved welfare practices) at the local commercial age (54 days). Tissue samples from abdominal and subcutaneous adipose tissue, breast, thigh, and liver were taken and stored at −20°C till the required lipids and fatty acid composition are analyzed.

### Lipid composition and fatty acids profile determination

According to Folch et al. [26], lipid samples were extracted; briefly using a sample of 5 g of the studied tissues, centrifuged, and homogenized in chloroform/methanol (2:1 *v/v*) solution. The homogenates were mixed and decanted, then chloroform-containing phase was collected, evaporated in a Rotavapor, and dried in an oven. Afterward, the percentage of lipids was calculated using the recuperation balloon’s weights.

Lipid extract aliquots were esterified with methanol (Joseph and Ackman, 1992), and the fatty acid composition of each aliquot has been identified using a Hewlett Packard 6890 gas chromatography. Fatty acid retention times and their peak surface areas (PSA) were automatically measured by an integrated computing Hewlett Packard. Individual fatty acids quantification was carried out by transformation and normalization of the PSA to milligrams % of the studied portions [27,28].

### Statistical analyses

Collected data in this randomized work were subjected to variance analysis [29]. Duncan’s multiple range test was used to distinguish treatment means. A single degree of freedom contrasts was adopted to estimate the significant impacts of the thermal acclimation and the linseed dietary supplementation. The level of *p* < 0.05 was considered for significance.

## Results and Discussion

### Total lipids (TL)

Lipid content was significantly (*p *< 0.05) altered by TC in subcutaneous adipose tissue and breast, showing a substantial increase in the thermally conditioned chickens independently of the linseed supplementation. It has increased by 38.1% versus 28.23% in control compared to the linseed-fed group. The breast total lipid contents were 11.63%, 11.13%, and 8.25% in AcL and Ac compared to C groups. The highest lipid deposition was noted in Ac birds (47%) (Table 1).

However, these results showed that TC and linseed supplementations are significantly interacted (*p* < 0.05). On the other hand, dietary linseed affected the lipid contents considerably in subcutaneous adipose tissue and abdominal fat (*p* < 0.05), leading to a reduction in their percentage in both control and Ac chickens. The fat content in the subcutaneous adipose tissue was 25.9% lower in the chickens that did not go through TC. On the contrary, the fat content decreased by 28.64% in the conditioned birds after feeding the linseed supplemented diet. The abdominal adipose tissue displayed a significant reduction in control as well as in Ac groups.

### Breast and thigh muscles’ fatty acids profiles

Higher content of SFA was noted in the C group compared to early- age- acclimated (basal diet) (AC), AcL, and AC groups. The SFA content was higher in the C group compared to other groups (*p* < 0.05). The total amounts of SFA, MUFA, and PUFA in the breast have been affected only in linseed diet supplementation (Table 2).

Combined acclimation and linseed supplementation were associated with decreased C14:0 and C16:0 content for muscles breast and thigh. As seen from the results, the incorporation of C18:3 varied between two traits. In the breast, the content of C18:3 was 2.08% in the CL group and 0.72% in the C group. In the thigh, the percentage varied between 5.31% in the Ac group and 2.68% in the C group. Eicosapentaenoic acid showed similar content, while docosahexaenoic acid was incorporated preferentially in the breast.

### Abdominal fats, liver, and subcutaneous fatty acids composition

TC of the birds positively affected the fatty acid profile of the liver, leading to a reduced content of C14:0 and SFA while increasing the content of C22:5 n-−3 and C22:5 n-−6, and PUFA. The same effect was observed in response to the linseed in the diet, which led to considerable augmentation of the MUFA (Table 3).

Early-age TC did not affect to a great extent the abdominal adipose tissue composition, while the opposite was observed for the subcutaneous. This corresponded to the results obtained for the lipid content of these tissues. More pronounced was the effect of the linseed in the diet, leading to a decrease in the total content of SFA while increasing the amount of MUFA and PUFA. The increased amount of MUFA in chickens’ adipose tissues contradicts the results observed for the liver and thighs in this study, indicating a depot-specific activity of the Δ9-desaturase [30].

## Discussion

The dietary approach can modify the poultry meat composition, where feedstuff ingredients, such as fat and oil, can be incorporated [24,27]. In our study, increased fat deposition in subcutaneous and abdominal tissues in heat-stressed animals was found, which might be explained by reducing thyroid hormone (T3) levels caused by hot temperatures [31]. Consequently, this reduces the basal metabolic rate and the animals’ physical activities, therefore leading to a redirected extra available energy stored as adipose tissues [32].

**Table 1.** Total lipid content (%) in various carcass parts of early-age acclimated broilers fed linseed supplemented diets.

Fat depot	Treatment			
	C	CL	Ac	AcL
Subcutaneous	38.1 ± 2.7^c^	28.23 ± 3.11^d^	47.66 ± 1.17^b^	34.01 ± 2.58^c^
Abdominal	59 ± 4.11^a^	53.22 ± 5.5^b^	64.12 ± 6.3^a^	51.25 ± 1.25^b^
Breast	8.25 ± 0.25^f^	9.2 ± 5.2^f^	11.13 ± 2.88^e^	11.63 ± 3.9^e^
Thigh	12.33 ± 2.8^e^	10 ± 1.4^f^	10.01 ± 2.6^f^	12. ± 3.5^e^
Liver	19 ± 0.25^d^	14.47 ± 1.2^e^	47 ± 2.5^b^	25.11 ± 2.9^d^

C = control (basal diet); AC = early-age acclimated (basal diet); Cl = fed 5% ground linseed; AcL = early-age acclimated and fed 5% ground linseed.

(*n* = 20) Means within a row without the same superscripts differ significantly (*p* < 0.05).

**Table 2.** Effects of thermal acclimation and linseed dietary inclusion on the fatty acid composition of breast and thigh muscles meat in broilers.

		Groups			
		C	CL	Ac	AcL
C14:0	Breast	0.60 ± 0.06^e^	0.47 ± 0.12^e^	0.58 ± 0.08^e^	0.52 ± 0.02^e^
	Thigh	0.52 ± 0.14^e^	0.22 ± 0.02^e^	0.31 ± 0.09^e^	0.13 ± 0.03^e^
C16:0	Breast	22.52 ± 0.74^a^	09.12 ± 1.26^c^	12.3 ± 0.3^b^	9.52 ± 1^c^
	Thigh	24.52 ± 0.74^a^	11.93 ± 1.26^b^	14.3 ± 1.3^b^	9.82 ± 1^c^
C18:0	Breast	7.39 ± 0.87^b^	6.62 ± 0.77^c^	6.67 ± 0.73^c^	7.99 ± 1.48^b^
	Thigh	9.67 ± 1.54^a^	5.02 ± 0.76^d^	7.28 ± 0.34^b^	5.18 ± 0.64^d^
C16:1	Breast	0.98 ± 0.31^c^	1.32 ± 0.38^b^	1.96 ± 0.24^a^	1.34 ± 0.54^b^
	Thigh	1.01 ± 0.27^c^	0.98 ± 0.54^c^	1.08 ± 0.30^c^	0.93 ± 0.23^c^
C18:1 *n*−9	Breast	39.02 ± 0.79^c^	42.12 ± 0.92^a^	39.59 ± 1^c^	43.26 ± 2.62^a^
	Thigh	41.61 ± 1.86^b^	39.98 ± 3.83^c^	38.54 ± 1.15^c^	40.73 ± 1.72^b^
C18:2 *n*−6	Breast	12.14 ± 0.99^c^	13.92 ± 1.08^b^	12.02 ± 0.92^c^	13.88 ± 1.12^b^
	Thigh	12.81 ± 1.95^c^	15.2 ± 1.24^a^	14.2 ± 1.24^a^	14.43 ± 1.36^a^
C18:3 *n*−3	Breast	0.72 ± 0.18^d^	2.08 ± 0.47^c^	1.08 ± 0.29^d^	2.09 ± 0.53^c^
	Thigh	2.68 ± 0.79^c^	6.02 ± 0.93^a^	5.31 ± 0.88^b^	6 ± 0.99^a^
C20:4 *n*−6	Breast	2.34 ± 0.34^b^	3.19 ± 0.92^a^	2.84 ± 0.95^b^	3.01 ± 0.57^a^
	Thigh	2.03 ± 0.76^b^	3.83 ± 0.94^a^	1.89 ± 0.99^b^	3.62 ± 1.37^a^
C20:5 *n*−3	Breast	3.33 ± 0.40^b^	4.23 ± 0.46^a^	3.86 ± 1.09^b^	4.79 ± 0.73^a^
	Thigh	1.42 ± 0.61^c^	4.22 ± 0.73^a^	3.62 ± 0.12^b^	4.01 ± 0.41^a^
C22:5 *n*−6	Breast	1.52 ± 0.56^b^	2.45 ± 0.58^a^	1.71 ± 0.43^b^	2.36 ± 0.49^a^
	Thigh	1.08 ± 0.08^b^	1.01 ± 0.02^b^	0.98 ± 0.41^b^	1 ± 0.22^b^
C22:5 *n*−3	Breast	0.04 ± 0.07^c^	1.04 ± 0.37^a^	0.06 ± 0.01^c^	0.62 ± 0.02^b^
	Thigh	0.08 ± 0.01^c^	1.36 ± 0.31^a^	0.98 ± 0.32^b^	1.39 ± 0.25^a^
C22:5 *n*−6	Breast	1.52 ± 0.56^b^	2.45 ± 0.58^a^	1.71 ± 0.43^b^	2.36 ± 0.49^a^
	Thigh	1.08 ± 0.08^c^	1.01 ± 0.02^c^	0.98 ± 0.41^c^	1 ± 0.22^c^
C22:5 *n*−3	Breast	0.04 ± 0.07^d^	1.04 ± 0.37^a^	0.06 ± 0.01^d^	0.62 ± 0.02^c^
	Thigh	0.08 ± 0.01^d^	1.36 ± 0.31^a^	0.98 ± 0.32^a^	1.39 ± 0.2^5a^
C22:6 *n*−3	Breast	0.91 ± 0.32^b^	1.6 ± 0.74^a^	0.73 ± 0.19^b^	1.8 ± 0.77^a^
	Thigh	0.06 ± 0.01^d^	0.32 ± 0.14^c^	0.28 ± 0.10^c^	0.3 ± 0.08^c^
*n*−6	Breast	16.22 ± 0.94^d^	19.87 ± 1.14^a^	17.08 ± 0.95^c^	19.35 ± 0.55^b^
	Thigh	15.94 ± 1.74^d^	20.08 ± 0.95^a^	17.18 ± 1.42^c^	19.52 ± 1.58^b^
*n*−3	Breast	5.04 ± 1.02^c^	9.01 ± 0.77^b^	6.22 ± 0.43^c^	9.6 ± 1.03^b^
	Thigh	4.28 ± 0.71^c^	11.99 ± 1.88^a^	10.29 ± 1.45^a^	11.82 ± 1.88^a^
*n*−6/*n*−3	Breast	3.29 ± 0.50^a^	2.2 ± 0.13^b^	2.74 ± 0.15^b^	2.02 ± 0.16^b^
	Thigh	3.74 ± 0.22^a^	1.69 ± 0.19^c^	1.67 ± 0.09^c^	1.66 ± 0.13^c^
SFA	Breast	31.42 ± 1.1^a^	19.32 ± 1.1^d^	29.86 ± 1.8^a^	19.73 ± 0.9^d^
	Thigh	34.94 ± 2.06^a^	25.9 ± 2.05^c^	30.09 ± 2.1^a^	25.95 ± 1.97^c^
PUFA	Breast	23.27 ± 1.05^b^	29.2 ± 0.9^a^	24.22 ± 1.05^b^	29.55 ± 0.96^a^
	Thigh	21.26 ± 1.2^b^	32.14 ± 1.1^a^	28.89 ± 1.69^a^	32.29 ± 2.18^a^

C = control (basal diet); AC = early- age- acclimated (basal diet); Cl = (fed 5% ground linseed); AcL = (early-age acclimated and fed 5% ground linseed).

(*n* = 20) Means within a row without the same superscripts differ significantly (*p* < 0.05).

**Table 3.** Effects of thermal acclimation and linseed dietary inclusion on broilers’ fatty acid composition of liver, subcutaneous, and abdominal fats of chickens.

		Groups			
		CL	C	Ac	AcL
C14:0	Subcutan	0.49 ± 0.09^e^	0.61 ± 0.06^e^	0.52 ± 0.04^e^	0.52 ± 0.08^e^
	Liver	3.6 ± 0.86^d^	6.21 ± 0.94^a^	6.42 ± 0.55^a^	0.67 ± 0.09^e^
	Abdominal	3.02 ± 0.79^d^	6.62 ± 0.88^a^	5.6 ± 1.26^b^	4.3 ± 0.91^c^
C16:0	Subcutan	20.43 ± 0.37^c^	26.32 ± 0.61^a^	18.78 ± 0.87^c^	18.63 ± 0.92^c^
	Liver	20.02 ± 1.13^c^	23.72 ± 1.664^b^	22.31 ± 1.43^b^	19.41 ± 1.31^c^
	Abdominal	4.31 ± 0.23^g^	7.6 ± 1.3^f^	8.01 ± 0.91^f^	10.92 ± 0.98^d^
C18:0	Subcutan	6.42 ± 0.58^d^	6.822 ± 0.64^d^	6.31 ± 0.39	7.36 ± 0.49
	Liver	6.39 ± 1.29^d^	5.8 ± 1.50^e^	4.92 ± 1.12^e^	5.3 ± 1.2^e^
	Abdominal	8.52 ± 1.55^c^	10.82 ± 0.87^a^	8.62 ± 1.6^e^	9.62 ± 1.3^b^
C16:1	Subcutan	0.86 ± 0.20^b^	0.42 ± 0.02^b^	0.39 ± 0.02^b^	0.72 ± 0.05^b^
	Liver	3.86 ± 0.93^a^	5.29 ± 0.73^a^	5.18 ± 0.76^a^	4.99 ± 1.08^a^
	Abdominal	0.82 ± 0.12^b^	0.6 ± 0.1^b^	0.72 ± 0.1^b^	0.78 ± 0.13^d^
C18:1 *n*−9	Subcutan	38.24 ± 0.97^a^	32.36 ± 1.10^a^	34.82 ± 0.67^a^	39.09 ± 0.81^a^
	Liver	6.82 ± 0.89^i^	6.72 ± 0.52^i^	7.8 ± 0.678^i^	5.3 ± 0.44^i^
	Abdominal	30.01 ± 1.59^g^	25.32 ± 1.39^h^	29.36 ± 1.514^g^	31.02 ± 1.75^g^
C18:2 *n*−6	Subcutan	16.38 ± 0.65^b^	12.32 ± 1.16^f^	13.78 ± 1.42^e^	17.01 ± 0.74^b^
	Liver	19.99 ± 0.74^a^	16.72 ± 1.47^b^	16.01 ± 0.92^b^	20.82 ± 1.67^a^
	Abdominal	15.08 ± 1.30^c^	14.6 ± 1.59^c^	15.74 ± 1.11^b^	14.78 ± 1.39^d^
C18:3 *n*−3	Subcutan	6.61 ± 0.67^e^	0.86 ± 0.2^i ^	2.61 ± 0.54^i^	5.89 ± 1.17^e^
	Liver	7.62 ± 1.3^d^	4.45 ± 0.85^f ^	5.62 ± 1.65^e^	6.6 ± 1.33^e^
	Abdominal	9.62 ± 0.83^b^	6.2 ± 1.12^e^	8.21 ± 1.12^c^	10.2 ± 1.35^a^
C20:4 *n*−6	Subcutan	2.73 ± 0.53^c^	1.89 ± 0.52^d^	2.09 ± 0.19^c^	2.98 ± 0.48^c^
	Liver	8.01 ± 0.89^a^	6.82 ± 1.14^b ^	7.87 ± 1.53^a^	5.48 ± 1.51^b^
	Abdominal	2.8 ± 0.86^c^	1 ± 0.30^d^	1.08 ± 0.28^d ^	2.7 ± 1.39^c^
C20:5 *n*−3	Subcutan	2.01 ± 0.37^b ^	1.92 ± 0.79^b ^	1.68 ± 0.47^c^	2.19 ± 0.48^b^
	Liver	3.41 ± 0.57^a ^	1.05 ± 0.23^c ^	1.62 ± 0.48^c^	2.66 ± 0.64^b^
	Abdominal	1.01 ± 0.21^b^	0.38 ± 0.18^d^	0.02 ± 0.01^d^	2.01 ± 0.77^b^
C22:5 *n*−6	Subcutan	0.09 ± 0.07^d^	0.01 ± 0.002^d^	0.03 ± 0.007^d^	0.08 ± 0.01^d^
	Liver	0.02 ± 0.001^d^	0.2 ± 0.01^c^	0.3 ± 0.04^c^	0.24 ± 0.02^c^
	Abdominal	1.01 ± 0.10^a^	0.82 ± 0.26^b^	0.08 ± 0.01^e^	1.6 ± 0.27^a^
C22:5 *n*−3	Subcutan	0.72 ± 0.03^d^	0.23 ± 0.02^d^	0.34 ± 0.04^d^	0.93 ± 0.11^c^
	Liver	2.04 ± 0.36^a^	1.01 ± 0.14^c^	1.99 ± 0.74^b^	2.8 ± 0.75^a^
	Abdominal	2.3 ± 0.46^a^	1.29 ± 0.5^c^	2.01 ± 0.52^a^	2.8 ± 1.19^a^
C22:6 *n*−3	Subcutan	0.36 ± 0.11^c^	0.18 ± 0.07^c^	0.22 ± 0.05^c^	0.29 ± 0.10^c^
	Liver	1.82 ± 0.25^a^	0.9 ± 0.07^b^	1.02 ± 0.21^b^	2.68 ± 1.13^a^
	Abdominal	1.99 ± 0.66^a^	1.01 ± 0.03^b^	1.09 ± 0.01^b^	1.89 ± 0.49^a^
*n*−6	Subcutan	20.26 ± 0.51^b^	14.32 ± 0.45^c^	16.83 ± 1.16^b^	20.18 ± 0.36^b^
	Liver	28.31 ± 1.35^a^	24.1 ± 1.47^a^	25.8 ± 0.87^a^	27.64 ± 1.09^a^
	Abdominal	21 ± 1.01^b^	17.43 ± 1.66^b^	17.02 ± 0.89^b^	19.78 ± 1.93^b^
*n*−3	Subcutan	10 ± 0.55^c^	3.28 ± 0.35^d^	4.97 ± 1.01^d^	9.42 ± 0.55^c^
	Liver	15.01 ± 0.95^b^	7.8 ± 0.89^c^	10.82 ± 0.96^c^	14.98 ± 1.02^b^
	Abdominal	15.13 ± 1.13^b^	9.86 ± 1.04^c^	11.98 ± 1.67^c^	17.01 ± 1.55^a^
*n*−6/*n*−3 ratio	Subcutan	2.02 ± 0.12^c^	4.39 ± 0.38^a^	3.46 ± 0.51^b^	2.14 ± 0.1^c^
	Liver	1.88 ± 0.04^c^	3.1 ± 0.21^b^	2.38 ± 0.13^c^	1.84 ± 0.05^c^
	Abdominal	1.38 ± 0.03^c^	1.76 ± 0.07^c^	1.43 ± 0.16^c^	1.16 ± 0.09^c^
SFA	Subcutan	27.51 ± 0.5^c^	42.98 ± 0.8^a^	34.38 ± 0.3^b^	28.18 ± 0.3^c^
	Liver	27.98 ± 1.6^c^	34.892 ± 1.8^b^	29.92 ± 1.01^c^	24.72 ± 1.39^d^
	Abdominal	29.98 ± 1.98^c^	37.93 ± 1.9^a^	34.99 ± 1.4^b^	30.04 ± 1.09^c^
PUFA	Subcutan	32.61 ± 0.6^c^	19.82 ± 0.97^d^	25.63 ± 0.69^c^	31.75 ± 0.68^c^
	Liver	44.78 ± 1.1^a^	34.41 ± 0.9^c^	38.44 ± 1.18^b^	46.99 ± 1.52^a^
	Abdominal	37.6 ± 1.2^b^	32.15 ± 0.9^c^	33.04 ± 1.02^c^	37.88 ± 1.56^b^

C = control (basal diet); AC = early -age- acclimated (basal diet); Cl = (fed 5% ground linseed); AcL = (early-age acclimated and fed 5% ground linseed).

(*n* = 20) Means within a row without the same superscripts differ significantly (*p* < 0.05).

TL and triglycerides decreased significantly by heat stress, whereas liver cholesterol content increased [10,32,33]. These findings support other studies conducted by Wang et al. [34] and Fu et al. [35]. However, our research found that rearing broilers in hot climate conditions with a diet supplemented with linseed improved the heat-related stress factors to control levels by affecting lipogenesis genes in chicken liver during the early-age acclimation. These findings also support many studies on mammals using essential fatty acids such as linoleic and linolenic acids [36-38].

The fatty acid profile is an essential quality trait of meat, closely related to its nutritional and healthy value. Although known for its high dietetic quality, poultry meat is subjected to various experiments to improve its fatty acid composition. As external factors, TC and linseed-supplemented diet influenced individual fatty acids profile in chicken breasts and thigh muscles and their total amounts. For muscles (breasts and thighs), linseed supplementation and acclimation correlated with decreased meristic and palmitic acid contents. It is known that these two fatty acids are hypercholesterolemic, and their high content in the diet might increase the risk of cardiovascular diseases. Hence, it could be suggested that acclimation and linseed supplementation have positively influenced broiler meat fatty acid composition.

Ibrahim et al. [38] and Kumar et al. [39] observed reduced content of SFA, which is in line with our results; however, they found a reduced MUFA level, which differed from our results. Similar to our findings, Leskovec et al. [40] and Nasir et al. [41] found better PUFA content in chickens fed linseed oil. Our results illustrate and confirm the possibility of the inclusion of linseed in the diet as a nutritional factor affecting the nutritional quality of poultry meat. The different influences of the factors and their interaction in the breast and thigh suggests that it might depend on the muscle type [42]. 

It was reported that a vital breast deposition of PUFA had been reported [43,44] differentiating in tissue FA profiles. This could have resulted from either their different roles in these tissues or their other phospholipid contents, as PUFA was incorporated preferentially into breast muscle phospholipids. As chicken cannot synthesize essential fatty acids, dietary sources will be the only alternative way. However, their meat incorporation depends on their dietary existence and their tissue oxidation metabolism rate. The breast meat alpha-linoleic acid was found to be higher in the CL group, while eicosapentaenoic acid was higher in the AcL group relative to the other fat groups (*p *< 0.05).

In this experimental work, proportions of SFA and PUFA were different, but no significant influence on MUFA was noted among broiler meat portions, as supported by Smink et al. [45]. The liver regulates the synthesis of MUFA according to Nasir et al. [41]; the increased content of dietary PUFA inhibits Δ^9^-desaturase activity in the liver, leading to a lower conversion of SFA to MUFA, which coincides with our results. On the other hand, another study [46] found that diet has limited liver fatty acid composition alteration. Shahid et al. [47] reported high PUFA C18:2 and C18:3 in heat-exposed birds, which is in line with our results.

Hepatic endogenous fatty acid synthesis could explain the decreased SFA effect as broilers fed sunflower and linseed showed the highest content of liver C18:0 and the lowest of C18:1 contents fatty acids. It shows the inhibitory action of PUFA on ∆^9^-desaturase activity [47]. Although dietary linseed decreased n-6 fatty acids while sunflower lowered the n-−3 content, both reflected the competition of these derivatives families by ∆^5^ and ∆^6^ desaturases. The same results in fat depots have been found by other authors [41,44,48].

The higher PUFA ratio of Ac broilers fed supplemented linseed oil might have resulted from their relatively higher lipogenic activity [47]. However, different fat deposition ratios of broilers supplemented with these diets could be the consequence of their preferences of fatty acid deposition, oxidation rates, or lipogenesis, explained at the transcriptional level, by regulatory networks of genes’ transcription and post-transcription that modulate lipid metabolism [49], which can be altered, in turn, by linseed-based diets [50].

## Conclusion

Although early-age acclimation of poultry chicks appeared to endow new physiological parameters, such as increased carcass adiposity at commercial age, reversed second treatment (dietary linseed inclusion) on lipid deposition was noted in various tissues in the edible parts. Moreover, early-age thermal acclimation of birds had influenced less fatty acid profile compared to linseed treatment. It was associated with a considerable decrease in the SFA and an increase in PUFA in birds. Except for liver and thigh meat, the content of MUFA was also increased in response to the linseed in the diet. Therefore, their combination might be an efficient method to improve animal thermoresistance and better meat quality. Further investigations are needed to assess current results focusing on the dependence of two treatments in modifying the more positively fatty acid composition of broiler meat.

## List of Abbreviations

AC: early-age acclimated (basal diet); AcL: early-age acclimated and fed 5% ground linseed; C: control (basal diet); Cl: fed 5% ground linseed; CLA: conjugated linoleic acid; ME: metabolizable energy; MUFA: monounsaturated fatty acid; PSA: peak surface areas; PUFAs: polyunsaturated fatty acids; SFA: saturated fatty acid; TC: thermal conditioning; TL: total lipids.
